# The Effect of the Anticonvulsant Topiramate on Acetylcholine‐Induced Calcium Signals is Linked to Sirt‐1 Activity

**DOI:** 10.1111/ejn.70514

**Published:** 2026-04-29

**Authors:** Marie‐Luise Kümmel

**Affiliations:** ^1^ Zoology/Physiology‐Neurobiology, ZHMB (Center of Human and Molecular Biology), Faculty NT‐Natural Science and Technology Saarland University Saarbrücken Germany; ^2^ Department of Neuroanatomy and Molecular Brain Research, Institute of Anatomy Ruhr University Bochum Bochum Germany

**Keywords:** Acetylcholine, Sirt‐1, Topiramate

## Abstract

Although some forms of epilepsy directly result from mutations in nicotinic acetylcholine receptors, none of the currently available antiepileptic drugs (AEDs) is specifically designed to target the cholinergic system. However, there is growing evidence that some established AEDs, which were primarily designed to modulate excitatory glutamatergic and/or inhibitory GABAergic currents, may also influence cholinergic signalling. This study therefore investigated whether topiramate (TPM), a second‐generation AED, directly affects calcium signals and whether the deacetylase sirtuin‐1 (Sirt‐1) contributes to this effect. Calcium imaging in the human neuroblastoma cell line SH‐SY5Y was used to quantify acetylcholine‐ and nicotine‐induced calcium signals following TPM treatment. To evaluate the role of protein deacetylases, TPM effects were further analysed in the presence of the deacetylase inhibitors trichostatin A (TSA) and inauhzin. TPM treatment significantly enhanced acetylcholine‐ and nicotine‐induced calcium signals. This effect of TPM was completely abolished in the presence of TSA. However, the presence of inauhzin resulted in an inhibitory effect of TPM on acetylcholine‐induced calcium signals. These findings reveal a previously uninvestigated modulatory effect of TPM on cholinergic calcium signalling that is directly dependent on the activity of deacetylases, like Sirt‐1. The results may contribute to a better understanding of TPM's anticonvulsive mechanisms of action.

## Graphical abstract

This work provides a potential new link between the anticonvulsive Topiramate, the deacetylase Sirt‐1 and the cholinergic signalling, suggesting that Topiramate may modulate cholinergic pathways in a Sirt‐1 depended manner.

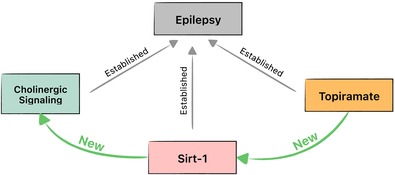

**Abbreviations**
ACh: acetylcholine
ADNFLE: autosomal nocturnal frontal lobe epilepsy
ADSHE: autosomal dominant sleep‐related hypermotor epilepsy
AED: antiepileptic drug
CV: coefficient of variation
FCS: foetal calf serum
GABA: gamma‐aminobutyric acid
In: inauhzin
JME: juvenile myoclonic epilepsy
nAChR: nicotinic acetylcholine receptor
Nic: nicotine
Pen/Strep: penicillin/streptomycin
PP: plateau potential
ROI: region of interest
Sirt‐1: sirtuin‐1
TPM: topiramate
TSA: trichostatin A

## 1 Introduction

Acetylcholine (ACh) is the second most abundant excitatory neurotransmitter in the human brain and plays a central role in memory formation, attention, arousal and sleep (Picciotto et al. 2012; Hasselmo 2006). Cholinergic neurons, located in the striatum, basal forebrain and brainstem, project to a broad spectrum of cortical and subcortical regions where they regulate presynaptic release of glutamate and GABA, the neuronal firing rate and the neuronal excitability (Picciotto et al. 2012; Ahmed et al. 2019). Various studies have shown that certain forms of epilepsy like ADNFLE (autosomal nocturnal frontal lobe epilepsy, renamed in 2016 to ADSHE, autosomal dominant sleep‐related hypermotor epilepsy, Tinuper et al. 2016) and JME (juvenile myoclonic epilepsy) are directly caused by gain‐of‐function mutations of nicotinic ACh receptors (Becchetti et al. 2023; Bertrand et al. 2002, 2005; Ghasemi and Hadipour‐Niktarash 2015). However, no therapeutics directly targeting cholinergic signal transmission are currently available, although different studies indicate that some commonly used anticonvulsant drugs that primarily affect glutamatergic or GABAergic signalling also modulate the cholinergic system. For example, carbamazepine, which induces seizure remission in nearly 70% of patients with ADFLE, was initially described as an inhibitor of voltage‐gated sodium channels and potentiator of GABAergic signals but was later found to also modulate ACh levels and act as an nAChR inhibitor (Ghasemi and Hadipour‐Niktarash 2015). Similar effects are seen with oxcarbazepine and lamotrigine (Ghasemi and Hadipour‐Niktarash 2015). Nevertheless, our understanding of this area remains very limited and precise mechanisms of action remain elusive. In the present study, we therefore investigated whether topiramate (TPM), a second‐generation antiepileptic drug (AED), modulates neuronal ACh‐induced calcium signals. While TPM is primarily known to target AMPA and GABA_A_ receptors as well as Na^+^‐ and Ca^2+^‐channels (Shank and Maryanoff 2008), some studies have shown that TPM modulates the cholinergic plateau potential (PP; Palmieri et al. 2000; Kuzmiski et al. 2005; D'Antuono et al. 2007), providing initial evidence that TPM may also affect the cholinergic system. The present study extends these findings by demonstrating for the first time that TPM enhances ACh‐ and nicotine‐induced calcium signals in the human neuroblastoma cell line SH‐SY5Y. SH‐SY5Y cells express native nicotinic acetylcholine receptors (nAChR) composed of ⍺3, ⍺5, ⍺7, β1 and β2 subunits in both RA‐differentiated and undifferentiated states (Warpman et al. 1998; Korecka et al. 2013), allowing a precise investigation of the effects of TPM. To further investigate the underlying mechanism of action, we focused on the protein deacetylase sirtuin‐1 (Sirt‐1), which exerts neuroprotective effects in epilepsy (Zeng et al. 2025) and increases the expression of the nicotinic acetylcholine receptor (nAChR) subunit α7 expression in the brain (Cao et al. 2020), making it a potential mediator of TPM's effects. This hypothesis was confirmed here by the impairment of the TPM‐induced effect on cholinergic signals in the presence of Sirt‐1 inhibitor inauhzin.

## 2 Methods

### 2.1 Cell Culture

Undifferentiated human neuroblastoma SH‐SY5Y cells were cultured in a 1:1 mixture of MEM + F12 cell culture media, supplemented with 10% FCS, 1% Pen/Strep, 0.5% GlutaMAX and 0.1% Gentamycin at 37°C and 5% CO_2_. One day before starting the experiments, cells were detached by using Trypsin/EDTA (0.05%) for 3 min at 37°C. Detachment was stopped by adding cell culture medium and washing once (3 min, 1100 ×*g*) in fresh cell culture medium. For calcium imaging experiments in a perfusion setup, 50,000 cells were plated on glass cover slips mounted onto a special perfusion silicon chamber (growing area: 20 × 4 mm). For stationary calcium imaging experiments, the same cell number was plated on 35 mm MatTek glass bottom dishes (14 mm microwell diameter, glass thickness No. 0).

### 2.2 Calcium Imaging

Cells were loaded with 2 μM Fluo‐4‐AM (0.2% DMSO in saline, Invitrogen) for 15 min, fluo‐4‐AM was washed off and cells were incubated for an additional 30 min to allow dye cleavage by intracellular esterases. During this time, cells were pretreated with the indicated drugs alone or in the following combinations: TPM (Cayman chemicals), Trichostatin A (TSA, Cayman chemicals) or TSA + TPM and Inauhzin (Sigma‐Aldrich) or Inauhzin + TPM. The treatment remained on the cells during the whole calcium imaging measurement. Calcium imaging was performed using either a stationary or a perfusion setup (indicated on every experiment). Each experiment was conducted exclusively under either perfusion or stationary conditions and analysis parameters were adapted accordingly to ensure comparability between the two setups. During the stationary calcium imaging setup, fluo‐4 loaded cells were recorded using a Visiscope spinning‐disc confocal system CSUW1 (Visitron) featuring a spinning disk unit CSU‐W1‐T2 and a sCMOS digital scientific grade camera (4.2 Mpixel rolling shutter version) on an inverted Nikon Ti‐E motorized microscope using a CFI P‐Fluor 20× objective (NA 0.5, WD = 2.10 mm). VisiView image acquisition software (Visitron) was used to capture pictures every second with exposure times of 500 ms, 30% laser intensity and 2 × 2 binning. Cells were recorded for 30 s before the manual application of ACh and 150 s thereafter. For the perfusion‐setup calcium imaging, a two‐channel pump with a constant perfusion rate of 6 mL/min was used. Cells were rinsed with saline solution plus the indicated drugs for 40 s before the stimulation to record the pre‐stimulation baseline. Cells were stimulated for 4 s with 5 μM acetylcholine‐chloride (ACh) or 10 μM nicotine (Nic) by changing the reservoir. The stimulation was followed by immediate washout. Fluorescence images were captured every 2 s (200 ms exposure) over 80 s using Leica DMIL LED microscope and QImaging Retiga R1 camera.

### 2.3 Data Analysis

Calcium imaging sequences were evaluated using ImageJ software. Cells were automatically detected using the ‘find foci’ plugin (Herbert et al. 2014) and individually marked as circular ‘regions of interest’ (ROI's) with a constant diameter of 12 pixels. For each cell, a relative calcium signal ratio (F_max_/F_min_; maximum fluorescence intensity after stimulation divided by minimum fluorescence intensity before stimulation) was calculated. To exclude cells from evaluation that show exceptionally high stimulation‐independent fluctuations in fluorescence intensity before stimulation (background signal), the coefficient of variation (CV; standard deviation/mean) was calculated for each cell before and after stimulation. In the stationary calcium imaging setup, cells were included in the analysis only if the CV was < 7% before stimulation and exceeded 7% after stimulation. In the perfusion setup, the corresponding threshold was 25%. To account for the heterogeneous nature of the SH‐SY5Y cell line—comprising substrate‐adherent (S‐type), neuroblastic (N‐type) and intermediate cells (I‐type) that differ morphologically and functionally (Bell et al. 2013)—calcium signals were analysed statistically at the individual single‐cell level rather than at the population level. Therefore, calcium signal ratios (F_max_/F_min_) were calculated from every single cell that was included for evaluation and statistically compared between treatments using the Mann–Whitney U‐test (*p* < 0.05). To analyse if a treatment affects the number of responding cells, the percentage of responding cells was calculated for each perfusion chamber or MatTek dish of each treatment group and statistically compared between treatments using the Mann–Whitney U‐test (*p* < 0.05). It was ensured that for each treatment, at least five perfusion chambers or MatTek dishes containing cells from at least three different passages were analysed, measured on at least two different experimental days. In the results section, the relevant sample sizes are reported as follows: the number of analysed cells for every treatment (‘cells_treatment_’), the number of measured chambers (‘chambers_treatment_’) and the number of experimental days (‘independent experiments’).

## 3 Results

### 3.1 TPM Increases ACh‐ and Nicotine‐Induced Calcium Signals

**FIGURE 1:**
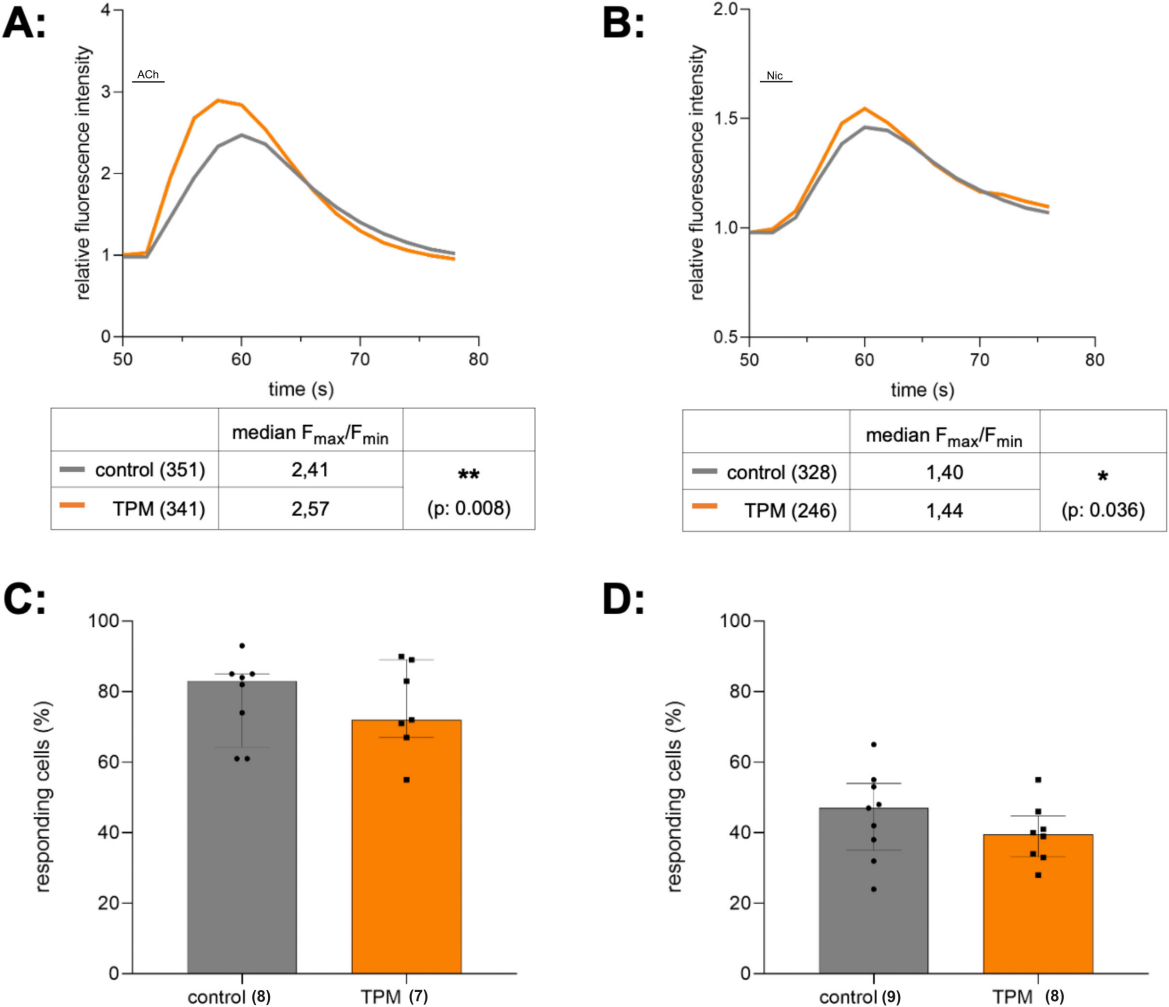
TPM enhances ACh‐ and nicotine‐induced calcium signals. (A, B) Time course of the relative fluorescence signal ratio (F/F_0_) of control or TPM‐treated cells right after stimulation with 5 μM ACh (A) or 10 μM Nicotine (B) in the perfusion‐system calcium system setup, plotted as median of all responding cells, number of analysed cells in brackets. Treatment with TPM increases ACh‐ and nicotine‐induced calcium signals (*p*: 0.008 and *p*: 0.036, respectively). Statistical analysis of F_max_/F_min_ ratio of every responding cell by using Mann–Whitney‐U‐Test. (C, D) Median percentage with IQR of cells responding to the ACh‐stimulus or Nic‐stimulus, chambers measured indicated as dots. TPM treatment does neither affect the number of cells responding to the ACh‐stimulus (C, *p*: 0.67) nor to the nicotine‐stimulus (D, *p*: 0.33).

To assess TPM's effect on the cholinergic signal transmission, calcium imaging was performed on human SH‐SY5Y neuroblastoma cells incubated with 30 μM TPM for 30 min before and during the measurement (Figure 1). Upon stimulation with 5 μM ACh, an agonist of nicotinic and muscarinic acetylcholine receptors, treatment with TPM did not affect the percentage of responding cells (Figure 1C, median percentage of responding cells control: 83%, TPM: 72%, U: 24, *p*: 0.67, chambers_control_: 8, chambers_TPM_: 7, two independent experiments), but significantly increased the calcium signal ratio (F_max_/F_min_) (Figure 1A, mean rank_control_: 326.58, mean rank_TPM_: 367.01, U: 52852, *p*: 0.008, cells_control_: 351, cells_TPM_: 341). To assess whether this effect is mediated via nicotinic acetylcholine receptors (nAChR), cells were stimulated with 10 μM nicotine (Nic), a specific nAChR agonist, instead of ACh. TPM similarly enhanced nicotine‐induced calcium signal ratio (F_max_/F_min_) (Figure 1B, mean rank_control_: 274.94, mean rank_TPM_: 304.27, U: 36218,5, *p*: 0.036, cells_control_: 328, cells_TPM_: 246) without affecting the total number of responding cells (Figure 1D median percentage of responding cells control: 47%, TPM: 39.5%, U: 25.5, *p*: 0.33 chambers_control_: 9, chambers_TPM_: 8, three independent experiments). However, nicotine stimulation resulted in a reduced number of responding cells and weaker calcium signals compared with ACh stimulation. Taken together, these results indicate that the treatment with TPM significantly affects neuronal ACh‐ and Nic‐induced calcium signals.

### 3.2 Inhibition of Deactylase Sirt‐1 Modulates TPM Effect on ACh‐Induced Calcium Signals

**FIGURE 2:**
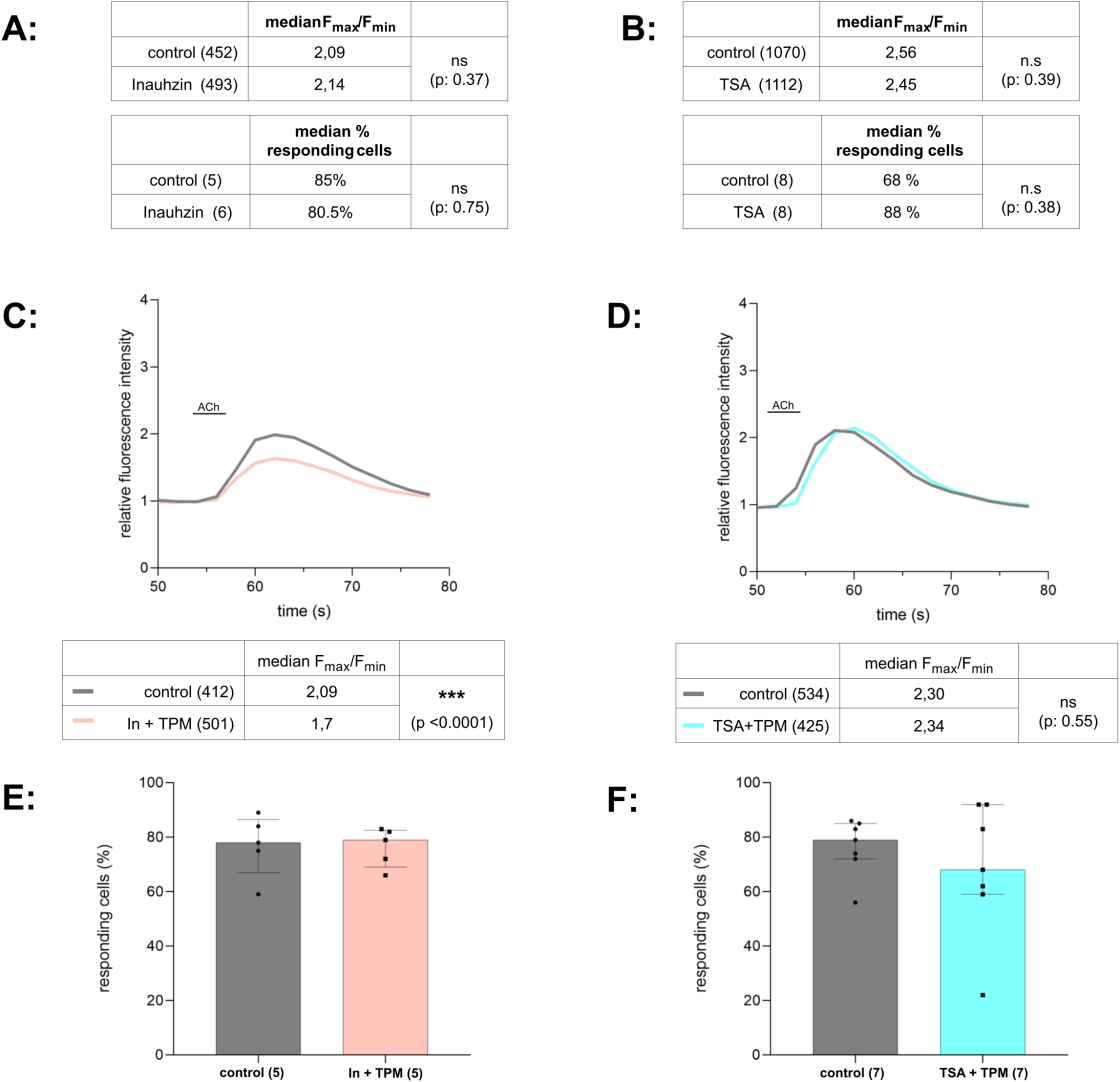
Inhibition of protein deacetylases with Inauhzin or TSA strongly affects TPM's effect on ACh‐induced calcium‐signals. (A) Cells were treated with Inauhzin (0.2 μM) alone for 30 min and ACh‐induced calcium signals in were evaluated using the perfusion‐system calcium imaging setup. Inauhzin does neither affect the total number of responding cells (*p*: 0.75) nor the ratio (F_max_/F_min_) of ACh‐induced calcium signals (*p*: 0.37). (B) Cells were treated with TSA (0.5 μM) alone for 30 min and ACh‐induced calcium signals were evaluated using the stationary calcium imaging setup. TSA does neither affect the total number of responding cells (*p*: 0.38) nor the ratio (F_max_/F_min_) of ACh‐induced calcium signals (*p*: 0.39). (C, D) Time course of the relative fluorescence signal (F/F_0_) of control (Inauhzin) vs. Inauhzin + TPM treated cells (C) or control vs. TSA + TPM treated cells (D) measured in the perfusion‐system calcium imaging setup. In the presence of Inauhzin, TPM exhibits an inhibiting effect on ACh‐induced calcium signals (C, *p* < 0.0001) whereas in the presence of TSA, no effect of TPM on ACh‐induced calcium signals is detectable (D, *p*: 0,55). (E, F) Median percentage with IQR of cells responding to the ACh‐stimulus after treatment with Inauhzin + TPM (E) or TSA + TPM (F) compared with control, single chambers measured indicated as dots, number of all measured cells in brackets. Neither the treatment with Inauhzin + TPM (E, *p*: 0.84) nor the treatment with TSA + TPM (F, *p*: 0.73) affects the percentage of responding cells.

It was previously shown that activation of histone deacetylase Sirt‐1 increases the expression of nAChR subunit α7 (Cao et al. 2020), suggesting a modulatory function of Sirt‐1 in cholinergic signalling. To assess if Sirt‐1 plays a role in the TPM signalling cascade leading to enhanced ACh‐induced calcium signals, the effect of TPM on ACh‐induced calcium signals was analysed in the presence of the specific Sirt‐1 inhibitor Inauhzin (In) (Zhang et al. 2012) (Figure 2A,C,E). To verify the overall role of deacetylases in the effect of TPM, we also examined TPM's effect in the presence of trichostatin A (TSA), a broad‐spectrum, mostly unspecific inhibitor of class I and II deacetylases (Kim et al. 2019) (Figure 2B,D,F). Analysis of the percentage of responding cells and the induced calcium signal ratios after treatment with 0.2 μM inauhzin or 0.5 μM TSA alone was performed to exclude any impact of these inhibitors on ACh‐induced calcium signals (Figure 2A,B). Neither Inauhzin nor TSA has any significant impact on the number of responding cells (median percentage of responding cells control: 85%, Inauhzin: 80.5%, U: 13, *p*: 0.75, chambers_control_: 5, chambers_inauhzin_: 6; median percentage of responding cells control: 68%, TSA: 88%, U: 23, *p*: 0.38, chambers_control_: 8, chambers_TSA_: 8) or on the ACh‐induced calcium signal ratios (Figure 2A,B, calcium signal ratios: mean rank_control_: 464.76, mean rank_inauhzin_: 480.55, U: 197695, *p*: 0.37, cells_control_ = 452, cells_Inauhzin_ = 493, two independent experiments; mean rank_control_: 772, mean rank_TSA_: 755, U: 283126, *p*: 0.45, cells_control_ = 1070, cells_TSA_: = 1112, three independent experiments).

Next, the effect of TPM on ACh‐induced calcium signals in the presence of either Inauhzin (In + TPM) or TSA (TSA + TPM) was investigated. As shown in Figure 2E,F, TPM did not affect the percentage of responding cells in the presence of inauhzin (Figure 2E, median percentage of responding cells control: 78%, Inauhzin + TPM: 79%, U: 11, *p*: 0.84, chambers_control_: 5 chambers_Inauhzin + TPM_: 5, two independent experiments) or TSA (Figure 2F, median percentage of responding cells control: 79%, TSA + TPM: 68%, U: 21.5, *p*: 0.73, chambers_control_: 7, chambers_TSA + TPM_: 7, two independent experiments). These results are consistent with those obtained after TPM incubation alone (Figure 1). However, an analysis of the ACh‐induced calcium signal ratios showed a significant modulation of TPM's effect by both protein deacetylase inhibitors (Figure 2C,D). In the presence of Inauhzin, TPM strongly inhibited ACh‐induced calcium signals (Figure 2C, mean rank_control_: 528.44, mean rank_inauhzin + TPM_: 398.25, U: 132641, Z: 7423, *p*: < 0.001, cells_control_: 412, cells_Inauhzin + TPM_: 501). This result is in direct contrast to the enhancing effect of TPM on ACh‐induced calcium signals observed in the absence of this inhibitor (compare with Figure 1). In the presence of TSA, the enhancing effect of TPM on ACh‐induced calcium signals is completely abolished (Figure 2D, mean rank_control_: 475,34, mean rank_TSA + TPM_: 485,86, U: 110984,5, Z: −0,584, *p*: 0.559, cells_control_534, cells_TSA + TPM_: 425). These results indicate that the activity of HDAC's, particularly the activity of Sirt‐1, plays a central role in the TPM signalling cascade.

## 4 Discussion

In this study, it was demonstrated that TPM directly impacts ACh‐ and nicotine‐induced calcium signals. This effect was modulated and abolished in the presence of deacetylase inhibitors, suggesting that the effect of TPM is linked to deacetylase activity.

In particular, the finding that TPM directly affects nicotine‐induced calcium signals provides important new insights into its mechanism of action, as previous studies have mainly focused on effects linked to the muscarinic system, such as a TPM‐induced depression of the cholinergic‐induced depolarizing PP in the hippocampal CA1 region and the subiculum (Palmieri et al. 2000; Kuzmiski et al. 2005; D'Antuono et al. 2007). The cholinergic‐induced PP refers to a prolonged depolarization of the membrane potential that occurs when depolarizing currents are injected in the presence of cholinergic agonists and depends on a functional interplay between activated muscarinic receptors and calcium influx through voltage‐gated calcium channels (Fraser and MacVicar 1996; Fraser et al. 2001). As a complement to these previous studies, the present study focused on nAChR‐mediated calcium dynamics and demonstrates a direct modulation of nicotine‐induced calcium signals in the presence of TPM, indicating that TPM affects not only the muscarinic system, as previously demonstrated by its effect on the depolarizing PP (Palmieri et al. 2000, Kuzmiski et al. 2005, D'Antuono et al. 2007) but also the nicotinic system. This is particularly interesting because epilepsy forms that are based on malfunctions of the cholinergic system specifically result from gain‐of‐function mutations in nicotinic receptors (Ghasemi and Hadipour‐Niktarash 2015; Picard et al. 1999). Furthermore, our results align with previous studies showing that many other anticonvulsive drugs commonly used in epilepsy therapy, like carbamazepine, lamotrigine or zonisamide, modulate nAChRs (for review, see Ghasemi and Hadipour‐Niktarash 2015). Interestingly, while many other AEDs like carbamazepine and lamotrigine mainly inhibit nAChR (Di Resta et al. 2010; Zheng et al. 2010), the present study demonstrates that TPM enhances ACh‐ and nicotine‐induced calcium signals. In this context, it should be noted that many previous studies investigated the effects of AEDs only on specific nAChR subtype compositions (e.g., ⍺_2_β_4_ (Di Resta et al. 2010) or ⍺_4_β_2_ (Di Resta et al. 2010; Zheng et al. 2010)) expressed in *Xenopus* oocytes or HEK293 cells. In contrast, the SH‐SY5Y cells used in the present study express multiple nAChR subunits, including ⍺3, ⍺5, ⍺7, β1 and β2 (Korecka et al. 2013). This is also particularly important for interpreting the differences in the percentage of responding cells and in the signal ratios between ACh‐ and nicotine‐induced calcium signals observed here. Different nAChR subtypes do not only differ in their sensitivity to ACh (e.g., potency of ACh when binding to recombinant ⍺_2_β_4_: 82 μM and ⍺_7_: 179 μM, Chavez‐Noriega et al. 1997) or nicotine (e.g., potency of nicotine on recombinant ⍺_2_β_4_: 20 μM and ⍺_7_: 113 μM, Chavez‐Noriega et al. 1997), but also in their desensitization dynamics (Paradiso and Steinbach 2003; Sokolova et al. 2005). Furthermore, the nicotine‐induced calcium signals arise from multiple sources: calcium enters directly through nAChRs, causing an initial depolarization and activation of voltage‐gated calcium channels (VGCCs). The calcium influx through nAChRs and VGCCs, in turn, induces the release from calcium from the endoplasmic reticulum (ER) (Shen and Yakel 2009). Therefore, further studies are required to determine which components of the nAChR‐mediated signalling cascade are predominantly affected by TPM. The abolishment of the enhancing effect of TPM in the presence of Sirt‐1 inhibitor inauhzin strongly indicates that the effect of TPM on ACh‐induced calcium signals might be directly linked to Sirt‐1 activity. Since the role of Sirt‐1 in the pathophysiology and treatment of epilepsy has been under discussion for many years (for review, see Zeng et al. 2025), the direct involvement of Sirt‐1 in TPM's signalling cascade provides new insights into this field of research. The hypothesis that deacetylases play a role in TPM's signalling cascade is also further supported by a study demonstrating that treatment of HeLa cells with TPM can lead to an inhibition of deacetylases and a hyperacetylation of histone H4 (Eyal et al. 2004). Clarification of the underlying signalling cascade and identification of the specific acetylcholine receptors subtypes involved in the effect could significantly contribute to developing more targeted treatments for epilepsy in the future.

## Author Contributions

**Marie‐Luise Kümmel:** conceptualization, data curation, formal analysis, investigation, methodology, resources, software, validation, visualization, writing – original draft, writing – review and editing.

## Funding

The author has nothing to report.

## Conflicts of Interest

The author declares no conflicts of interest.

## Data Availability

Data available on request from the authors. I confirm that I have read the Journal's position on issues involved in ethical publication and affirm that this report is consistent with those guidelines.
